# The *Devon Active Villages Evaluation* (DAVE) trial: Study protocol of a stepped wedge cluster randomised trial of a community-level physical activity intervention in rural southwest England

**DOI:** 10.1186/1471-2458-12-581

**Published:** 2012-08-01

**Authors:** Emma Solomon, Tim Rees, Obioha C Ukoumunne, Melvyn Hillsdon

**Affiliations:** 1 Sport and Health Sciences, College of Life and Environmental Sciences, University of Exeter, St. Lukes Campus, Heavitree Road, Exeter, EX1 2LU, United Kingdom; 2 PenCLAHRC, Peninsula College of Medicine and Dentistry, University of Exeter, Veysey Building, Salmon Pool Lane, Exeter, United Kingdom

**Keywords:** Physical activity, Stepped wedge cluster randomised trial, Community-level intervention, Rural communities

## Abstract

**Background:**

Although physical inactivity has been linked with numerous chronic health conditions and overall mortality, the majority of English adults report doing insufficient physical activity. To increase population physical activity levels, researchers have called for more community-level interventions. To evaluate these complex public health interventions, innovative study designs are required. This study protocol describes *Devon Active Villages*, a community-level intervention providing physical activity opportunities to 128 rural villages in southwest England, and the methods used to evaluate its effectiveness in increasing physical activity levels.

**Methods/Design:**

A stepped wedge cluster randomised trial will be used to evaluate whether *Devon Active Villages* leads to increased physical activity levels in rural communities. Community engagement will help tailor activity programmes for each village; communities will then be supported for a further twelve months. The intervention will be delivered over four periods, each lasting twelve weeks. Data collection consists of a postal survey of a random sample of adults aged 18 years and over, at baseline and after each of the four intervention periods. The questionnaire includes questions on participant demographics, physical activity behaviour, local environment characteristics, awareness of local activity programmes, and psychosocial factors. Based on detecting an increase in the proportion of people who meet physical activity guidelines (from 25% to 30%), at least ten respondents are needed from each of the 128 villages at each stage (80% power at the 5% level of significance). Anticipating a 20% response rate, 6,400 questionnaires will be sent out at each stage (i.e., 50 surveys to each village). Using data from all five periods, a comparison of study outcomes between intervention and control arms will be performed, allowing for time period (as a fixed effect) and the random effect induced by correlation of outcomes (clustering) within villages.

**Discussion:**

This paper describes the use of a stepped wedge cluster randomised trial to evaluate a complex, community-level physical activity intervention in an under-studied population of adults in rural communities in southwest England. The study addresses gaps in the current literature by providing new insights into physical activity levels in this population.

**Trial Registration Number:**

Current Controlled Trials ISRCTN37321160

## Background

In developed and many developing countries physical inactivity is one of the most important public health problems of the 21^st^ century [1]. There is strong evidence linking physical inactivity with various chronic conditions, such as coronary heart disease, stroke, type 2 diabetes, cancer, obesity and mental health problems [1-3], and physical inactivity has been identified as a leading risk factor for mortality, estimated to cause 6% of deaths globally [4]. In contrast, the numerous benefits of a physically active lifestyle have been well documented [3]. Despite the preceding evidence, in England only 29% of women and 39% of men report doing sufficient physical activity to meet the minimum recommended guidelines of 150 minutes of moderate intensity physical activity per week or 75 minutes of vigorous intensity physical activity per week [5]. This level of physical inactivity is estimated to cost the United Kingdom National Health Service £0.9 billion per year [6].

Substantial health benefits can be achieved through relatively modest changes in activity behaviour among large segments of the population [7], and therefore physical activity interventions are now considered to be as important to population health as other high profile interventions, such as those lowering tobacco use or reducing blood pressure [2]. Although the health benefits of physical activity are now well-established, little is known about the effectiveness of interventions designed to improve population physical activity [8]. The majority of physical activity interventions have been delivered at the level of the individual, aimed at changing personal behaviour [9]. To change population prevalence, interventions need to be effective, but they also need to reach large numbers of people. Although some individual-level interventions are effective, their reach is limited when compared with community-level interventions. It is community-level interventions that have the potential to produce long-lasting benefits for the whole community, but evidence as to which type of community-wide interventions are most effective is currently weak [10].

A recent review of research examining the effectiveness of community-level interventions to promote physical activity reported that many studies used weak evaluation designs, such as uncontrolled, pre-post evaluations, and could not attribute any observed changes to the intervention [10]. One example of a community-level intervention evaluation that did include control communities—but was non-randomised—was the ‘Cycling Demonstration Towns’ programme in England [11], in which the intervention involved town-wide media campaigns, personalised travel planning, cycle training, repair services, and cycling infrastructure improvements. A controlled, repeated cross-sectional study examined the effect of the intervention in six towns between 2005 and 2008 using telephone surveys of quota samples of local residents [11]. The average annual percentage increase in the number of cyclists on the road was 4%. Net increases were also found in the proportions of residents who reported cycling for at least 30 minutes on 12 or more days per month (0.97% or 1.65%, depending on the choice of control areas) [11].

Reviews of physical activity correlates suggest that a combination of personal, social and environmental factors are associated with physical activity prevalence [12], but there are very few evaluations of the effects of changes to either social or built environments, and studies of the built environment are almost exclusively restricted to urban environments [10,13]. Both urban and rural dwellings report similarly low levels of physical activity in adults: on average, 9.5 days per month (95% CI: 9.3-9.6) of moderate-to-vigorous intensity physical activity for at least 30 minutes [5]. Although 20% of the population live in non-urban dwellings [5], rural populations are generally understudied [13,14]. Additionally, access to recreational facilities and other environmental supports for physical activity (e.g., neighbourhood ‘walkability’, convenient access to destinations, and perceived safety) have been shown to be related to physical activity participation [15], with people in rural areas being more likely to report lack of facilities as a barrier to physical activity [16].

Randomised controlled trials are considered the most powerful tool in research design for evaluating interventions, due to their rigorous study design and strict randomisation procedures [17]. Traditional randomised controlled trials, where individual participants are randomised, are not always reproducible in the real world and tend to focus on individuals rather than communities, raising doubts about whether a subsequent scaling up of individual interventions to larger populations would lead to changes in population prevalence [18]. It has been suggested that when evaluating interventions that are by necessity delivered to groups rather than individuals, cluster randomised trials, which randomise groups (e.g., communities, villages, towns) and measure outcomes on individual participants within those groups, are more appropriate [9,19].

Cluster randomised trials commonly use a parallel group design, in which the clusters are randomised to either the intervention or control arm of the study. For practical reasons it is often not possible to deliver an intervention to many clusters at the same time. In addition, it is often regarded as unethical to withhold an intervention from a proportion of participants if it is believed that the intervention will do more good than harm. In these circumstances, stepped wedge trial designs [20], where the intervention is delivered sequentially to all trial clusters over a number of time periods, is an alternative to the traditional parallel groups design. In a stepped wedge design, clusters effectively cross over from the control to the intervention group. The stage at which the clusters cross over is randomised. Outcomes are measured on the study participants in all clusters at every time period so that each cluster provides data points in both the control and intervention conditions [21]. Examples of stepped wedge investigations include the efficacy of Hepatitis B vaccinations [22], the effect of housing improvements on respiratory health symptoms [23], and different tuberculosis treatments on number of disease episodes [24].

The objective of this paper is to describe the protocol of a stepped wedge cluster randomised trial for evaluating the effectiveness of a community-level intervention to increase physical activity in rural villages in southwest England. The intervention will identify community needs and then provide resources and support to initiate local activity programmes, ultimately aiming for the activities to become self-sustaining over time. The intervention is expected to improve physical activity participation after each village receives the intervention. It is also anticipated that changes will be observed in levels of social support, physical activity intentions, awareness and use of local facilities, and perceived village supportiveness of physical activity.

## Methods/Design

### Study design

**Figure 1:**
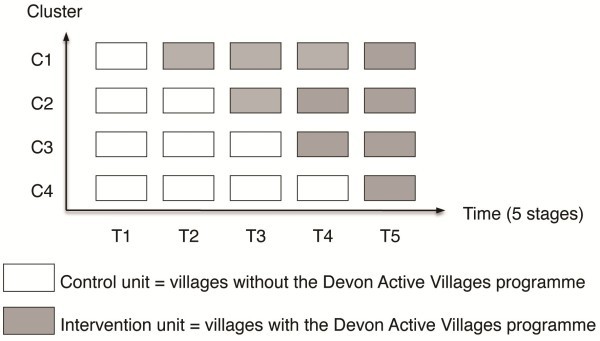
**Design of the DAVE study.** One cluster (C1, C2, C3 or C4) represents one group of intervention villages. Each time period (T1, T2, T3, T4, or T5) represents a data collection point. Each unit (control or intervention) represents one time period of one cluster.

The *Devon Active Villages Evaluation* (DAVE) protocol is based on a stepped wedge cluster randomised controlled trial design (Figure 1). During the DAVE study, the intervention will be rolled out sequentially to 128 rural villages (clusters) over four time periods. The evaluation will consist of data collection at five fixed time points (baseline and following each of the four intervention periods). The period in which the villages first receive the intervention will be randomly assigned, stratified by the seven regions of the county of Devon (see below). The intervention will be fully implemented by the end of the trial, with all 128 villages receiving the intervention: 22 first receiving the intervention at period 2, 36 at period 3, 35 at period 4, and 35 at period 5.

### Setting and participants

*Devon Active Villages* is a community-level intervention coordinated by *Active Devon,* the Devon county partnership for sport and physical activity. Active Devon received circa £950,000 funding for the Devon Active Villages intervention from Sport England (the government body for sports promotion) and Devon County Council as part of Sport England’s ‘Rural Communities’ funding scheme. The Devon Active Villages Evaluation (DAVE) research study is being conducted by the University of Exeter in close liaison with Active Devon.

Devon is characterised by ten distinct regions, of which three are urban (Exeter, Plymouth and Torbay), and seven are rural (East Devon, Mid Devon, North Devon, South Hams, Teignbridge, Torridge and West Devon). All intervention villages are located in one of the seven rural regions. The Devon Active Villages intervention will provide activities for all age groups.

In the initial planning of the intervention, Active Devon identified 155 rural villages to receive the Devon Active Villages intervention across the course of three years. Prior to the intervention, Active Devon ran a pilot intervention with 15 villages, the outcome of which was used to inform the main intervention protocol.

### Recruitment and randomisation

Of the remaining 140 villages that were not part of the pilot, twelve could not be included in the evaluation due to engagement with local community members before baseline data collection had commenced. Thus, the remaining 128 villages (clusters) were recruited and randomised to first receive the intervention in one of the four periods, stratified by region. Villages with populations of 500–2000 people formed the sampling frame for the intervention. These population boundaries were set so that villages were large enough to have local facilities suitable for physical activity, but limited in the amount of activity opportunities they offered.

Data collection for the evaluation study will focus on adults aged 18 years and over. The study will use a repeated cross-sectional design, in which a random sample of people within each cluster will be surveyed at each stage. A complete list of all households in each of the 128 study villages will be obtained using the Postcode Address File (Address List Utility, Arc en Ciel, Version 3.1 PAF Quarter 1, 2011). The order in which households are approached to participate in the survey at each stage will be randomly generated. One adult per household will be randomly selected. If there are multiple eligible adults in the household, an invitation to complete the survey will be given to the adult who has most recently had a birthday.

### Intervention

The primary objective of the Devon Active Villages intervention is to improve participation in physical activity by offering people of all ages increased opportunities to experience the enjoyment of sport and physical activity. The intervention will be implemented and coordinated locally by Local Delivery Partners. Local Delivery Partners include District Authority Sports Development Teams and community-based charitable organisations, some of which manage local facilities as well as maintain and develop activity opportunities in the local area. Each Local Delivery Partner will deliver the intervention in one of the seven regions. It was necessary to have different Local Delivery Partners for each area due to the large number of villages receiving the intervention in each period, and because the villages are spread across the whole county. No one Local Delivery Partner is of sufficient size to cover the whole county. Each Local Delivery Partner is given strategic support from Active Devon as well as a clear framework and timescales around the delivery of the intervention with strong focus on generating a local needs led approach to designing the activities.

**Figure 2:**
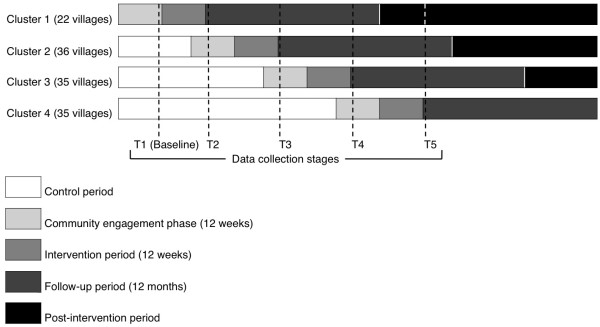
Data collection timeline for the Devon Active Villages Evaluation study.

Each village will receive a ‘community engagement phase’ for twelve weeks prior to the intervention (Figure 2). During this phase, Local Delivery Partners will engage with the local people, elected member structures, schools and other community groups to carry out a local needs assessment, an assessment of the activities currently on offer, and the activities’ take-up and capacity. This will often include, but not limited to, people being directly surveyed to find out what activities they would like the Devon Active Villages programme to provide.

The programme will then deliver twelve weeks of physical activity sessions, with each village receiving at least three different types of activities. These activity sessions will be subsidised using programme funds. Local Delivery Partners will coordinate delivery of the intervention by finding suitable activity venues, purchasing necessary equipment and hiring local experts to deliver the activities. Community volunteers will also be recruited to help run the activities and will be provided with mentoring support throughout the programme. Local Delivery Partners will advertise the Devon Active Villages activity sessions using local media (e.g., newspapers, posters, leaflets, village newsletters).

Each village will also be supported for twelve months following the intervention, when Local Delivery Partners will help the communities to sustain the intervention activities, by providing specialist support, regular mentoring for the volunteers and additional funding or equipment if necessary. Additionally, local people will be offered coaching qualifications to help the villages continue the activities independently.

### Outcome measurement

The primary analysis will compare the proportion of adults meeting recommended guidelines for the minimum level of physical activity (i.e., 150 minutes of moderate intensity physical activity per week or 75 minutes of vigorous intensity physical activity per week) between the intervention and control modes. Secondary outcomes will be social support, physical activity intentions, awareness and use of local facilities, perceived village supportiveness of physical activity, and awareness and participation in the Devon Active Villages intervention.

### Data collection

Postal questionnaires and participant information sheets will be sent out to participants at baseline (in the month prior to the first intervention period commencing) and within a week of each of the four intervention periods ending (Figure 2). The participant information sheet makes clear the voluntary nature of the study and therefore informed consent to participate in the study will be implied when participants return a completed questionnaire. If the number of completed questionnaires returned within three weeks of the initial mailing is insufficient, additional questionnaires will be sent out to new households. Participants will receive the questionnaire, a participant information sheet and a prepaid return envelope. It is possible that some individuals may receive the questionnaire on two or more occasions. In such cases, if returned, demographic variables (gender, age, height, weight) will be used to identify this wherever possible. These participants will remain in the analysis, but it will be recorded that each participant has completed the questionnaire on more than one occasion.

### Measures

#### Demographic characteristics

The survey will include questions on gender, age, height, weight, health, occupation, car ownership, children in the household, and dog ownership, based on questions from national surveys from different populations (e.g., Burton et al., [25], Craig et al., [5]).

#### Physical activity

Physical activity will be measured using the short version of the International Physical Activity Questionnaire (IPAQ) [26]. The IPAQ short-form consists of questions on the number of days and time spent on physical activity at moderate and vigorous intensity, as well as time spent walking and sitting. The mean values for each activity category will be calculated and expressed as metabolic equivalent (MET) minutes per week, and combined to categorise people into ‘low’, ‘moderate’ or ‘high’ activity classifications. The self-administered short-form IPAQ has been found to have acceptable levels of validity and reliability [26].

#### Local area

To assess perceived characteristics of the local environment a scale will be used that was initially developed for use in another United Kingdom health study. Participants are asked to rate their agreement with 12 items on factors such as aesthetics, green space, access to amenities, traffic, safety and convenience of routes. The scale has been found to have acceptable levels of test-retest reliability [27]. Questions on perceived proximity and use of different recreational facilities are also included. These items were previously found to have acceptable test-retest reliability [28].

#### Physical activity campaigns/programmes

The survey will contain questions on participants’ awareness of and participation in local physical activity campaigns. The survey will also ask about awareness of Devon Active Villages, participation in programme events, and opinions on the programme.

#### Psychosocial correlates

Participants will be asked about their intentions to be more active in the future. The survey will also ask them to rate the importance they place on physical activity on a scale from 0 (not at all) to 10 (very), as well as their physical activity confidence and the extent to which they are trying to do more activity [29]. Finally, a series of eight questions will ask participants to rate their agreement with statements about their physical activity habits, social norms, and perceived village supportiveness of activity. These questions were initially developed for use in an Australian cohort study (n = 2,485) [25].

### Sample size

To detect an increase from 25% to 30% in the percentage of people who meet guidelines for recommended physical activity levels, 10 participants need to be recruited from each of the 128 villages at each study period to achieve 80% power at the 5% significance level [30]. A recent pilot for a population study of travel behaviour in the United Kingdom achieved a response rate of 20% for a short questionnaire postal survey [31]. On this basis, 50 surveys will be sent out to each village at each stage, anticipating that we will obtain at least 10 responses per village per stage (20% response rate). This means that 6,400 surveys will be sent out at every stage with the expectation that at least 1,280 will be completed and returned. If this response rate is not achieved within three weeks of the surveys being posted, an additional five surveys will be sent out to extra households for every one survey missing (20% response rate).

### Statistical analysis

For any given outcome, data collected across all five periods will be used in a single analysis comparing the intervention and control modes. Analyses will use the intention-to-treat principle, with participants analysed according to the mode their village (cluster) was in for the stage at which they provided outcome data. Random effects (“multilevel”) linear regression models estimated using maximum likelihood [32] will be fitted to compare quantitative outcomes between the intervention and control modes, specifying the village effect as random; marginal logistic regression models using Generalised Estimating Equations (GEE) with information sandwich (“robust”) estimates of standard error specifying an exchangeable correlation structure [33] will be fitted to compare binary outcomes. Both the random effects model and GEE methods allow for correlation of outcomes within the same village cluster. Under both methods, a binary predictor variable will be used to indicate intervention versus control status and period of study, gender and age will be adjusted for. All analyses will be carried out using Stata software (StataCorp. 2011. *Stata Statistical Software: Release 12.* College Station, TX: StataCorp LP).

### Ethical consideration

The study received ethical approval from the Sport and Health Sciences Ethics Committee at the University of Exeter (February 2011).

## Discussion

This paper has outlined the Devon Active Villages Evaluation study design and data collection, as well as details on the implementation of the intervention. The DAVE study is the first to use a stepped wedge cluster randomised controlled trial design to evaluate the effectiveness of a community-level intervention designed to increase physical activity. The stepped wedge design is advantageous in studies where the intervention cannot be withheld from a proportion of the population and cannot be delivered to all intervention clusters at the same time. This study will demonstrate that it is possible to evaluate physical activity interventions using a stepped wedge trial design.

Strengths of the study will include the number of participating villages and the multiple data collection stages. The main limitation of the study is the self-reported outcome measure of physical activity that may lead to some misclassification. The implementation of the Devon Active Villages intervention may increase physical activity participation in rural villages in southwest England. The results from the study will contribute to the limited research available on physical activity in rural communities in England and other developed countries. This pragmatic evaluation of a community-led intervention is expected to provide a model of how to evaluate physical activity promotion in the community when it is being delivered by local organisations that frequently deliver such interventions with no evaluation at all. The study should help demonstrate how independent researchers and practitioners can successfully work together to evaluate natural experiments in real life settings.

In conclusion, the Devon Active Villages Evaluation study is believed to fill gaps in the current literature, providing new insights into rural physical activity, using innovative study designs to evaluate the intervention, and developing collaborations between researchers and practitioners to evaluate natural experiments. Therefore, the results from this study will contribute to the body of evidence on stepped wedge cluster randomised trials and community-level interventions, and may be useful for researchers and practitioners for future evaluations of complex public health interventions. In addition, if the Devon Active Villages intervention proves successful in improving population physical activity prevalence the intervention could be disseminated at national and international level.

## Competing interests

As part of an Economic and Social Research Council PhD CASE Studentship grant, the research is partially funded by Active Devon, but the research work and results are completely independent and not biased by the opinions of Active Devon.

## Authors’ contributions

The study chief investigators ES, TR and MH were responsible for identifying the research question, the design of the study, obtaining ethics approval and the acquisition of funding. OCU contributed to the fine-tuning of the methodology and statistical analysis. All authors helped draft and revise the manuscript and approved the final version.

## Pre-publication history

The pre-publication history for this paper can be accessed here:

http://www.biomedcentral.com/1471-2458/12/581/prepub

## References

[B1] World Health Organization Global Health Risks: Mortality and burden of disease attributable to selected major risks 2009 World Health Organization, Geneva, Switzerland

[B2] Department of Health Physical Activity, Health Improvement and Protection: Start Active, Stay Active: A report on physical activity from the four home countries’ Chief Medical Officers 2011 Department of Health, London

[B3] Physical Activity Guidelines Advisory Committee Physical Activity Guidelines Advisory Committee Report 2008 U.S. Department of Health and Human Services, Washington, DC 10.1111/j.1753-4887.2008.00136.x 19178654

[B4] World Health Organization Global Recommendations on Physical Activity for Health 2010 World Health Organization, Geneva, Switzerland 26180873

[B5] Craig R, Mindell J, Hirani V Health Survey for England 2008, Volume 1: Physical activity and fitness 2009 The NHS Information Centre, Leeds

[B6] Scarborough P Bhatnagar P Wickramasinghe KK Allender S Foster C Rayner M The economic burden of ill health due to diet, physical inactivity, smoking, alcohol and obesity in the UK: an update to 2006–07 NHS costs J Public Health 2011 10.1093/pubmed/fdr033 21562029

[B7] Haskell WL Lee IM Pate RR Powell KE Blair SN Franklin BA Macera CA Heath GW Thompson PD Bauman A Physical activity and public health: updated recommendation for adults from the American College of Sports Medicine and the American Heart Association Circulation 2007 116 1081 1093 17671237 10.1161/CIRCULATIONAHA.107.185649

[B8] Foster C Hillsdon M Thorogood M Interventions for promoting physical activity (Review) Cochrane Db Syst Rev 2005 2005 Issue 1 10.1002/14651858.CD003180.pub2 PMC4164373 15674903

[B9] House of Lords Science and Technology Select Committee: Behaviour Change 2011 Authority of the House of Lords, London

[B10] Baker PRA Francis DP Soares J Weightman AL Foster C Community wide interventions for increasing physical activity Cochrane Db Syst Rev 2011 2011 Issue 4 10.1002/14651858.CD008366.pub2 21491409

[B11] Sloman L Cavill N Cope A Muller L Kennedy A Analysis and synthesis of evidence on the effects of investment in six Cycling Demonstration Towns 2009 Department for Transport and Cycling England

[B12] Trost SG Owen N Bauman AE Sallis JF Brown W Correlates of adults' participation in physical activity: review and update Med Sci Sports Exerc 2002 34 1996 2001 10.1097/00005768-200212000-00020 12471307

[B13] Ogilvie D Griffin SJ Jones A Mackett R Guell C Panter J Jones N Cohn S Yang L Chapman C Commuting and health in Cambridge: a study of a 'natural experiment' in the provision of new transport infrastructure BMC Public Health 2010 10 703 10.1186/1471-2458-10-703 21080928 PMC2999608

[B14] Saelens BE Sallis JF Frank LD Environmental correlates of walking and cycling: findings from the transportation, urban design and planning literatures Ann Behav Med 2002 25 80 91 12704009 10.1207/S15324796ABM2502_03

[B15] Bauman A Bull F Environmental Correlates of Physical Activity and Walking in Adults and Children: A Review of Reviews 2007 National Institute of Health and Clinical Excellence (NICE), Loughborough

[B16] Brownson RC Housemann RA Brown DR Jackson-Thompson J King AC Malone BR Sallis JF Promoting physical activity in rural communities: Walking trail access, use, and effects Am J Prev Med 2000 18 235 241 10.1016/S0749-3797(99)00165-8 10722990

[B17] Sibbald B Roland M Understanding controlled trials: Why are randomised controlled trials important? Brit Med J 1998 316 201 10.1136/bmj.316.7126.201 9468688 PMC2665449

[B18] Sanson-Fisher RW Bonevski B Green LW D'Este C Limitations of the Randomized Controlled Trial in Evaluating Population-Based Health Interventions Am J Prev Med 2007 33 155 161 10.1016/j.amepre.2007.04.007 17673104

[B19] Craig P Dieppe P Macintyre SJ Michie S Nazareth I Petticrew M Developing and evaluating complex interventions: new guidance Medical Research Council 2008

[B20] Cook TD Campbell D Quasi-experimentation: Design and analysis issues for field settings 1979 Houghton Mifflin, Boston

[B21] Brown CA Lilford RJ The stepped wedge trial design: a systematic review BMC Med Res Methodol 2006 6 54 10.1186/1471-2288-6-54 17092344 PMC1636652

[B22] Gambia Hepatitis Study Group The Gambia Hepatitis Intervention Study Cancer Res 1987 47 5782 5787 2822233

[B23] Somerville M Basham M Foy C Ballinger G Gay T Shute P Barton AG From local concern to randomised trial: the Watcombe Housing Project Health Expect 2002 5 127 135 10.1046/j.1369-6513.2002.00167.x 12031053 PMC5060144

[B24] Grant AD Charalambous S Fielding KL Day JH Corbett EL Chaisson RE De Cock KM Hayes RJ Churchyard GJ Effect of routine Isoniazid preventative therapy on Tuberculosis incidence among HIV-infected men in South Africa J Amer Med Assoc 2005 22 2719 2725 10.1001/jama.293.22.2719 15941800

[B25] Burton NW Oldenburg B Sallis JF Turrell G Measuring psychological, social, and environmental influences on leisure-time physical activity among adults Aust N Z J Public Health 2007 31 36 43 10.1111/j.1753-6405.2007.00008.x 17333607

[B26] Craig CL Marshall AL Sjostrom M Bauman AE Booth ML Ainsworth BE Pratt M Ekelund U Yngve A Sallis JF Oja P International physical activity questionnaire: 12-country reliability and validity Med Sci Sport Exer 2003 35 1381 1395 10.1249/01.MSS.0000078924.61453.FB 12900694

[B27] Ogilvie D Mitchell R Mutrie N Petticrew M Platt S Perceived characteristics of the environment associated with active travel: development and testing of a new scale Int J Behav Nutr Phy 2008 5 32 10.1186/1479-5868-5-32 PMC2416440 18513430

[B28] Sallis JF Johnson MF Calfas KJ Caparosa S Nichols JF Assessing perceived physical environmental variables that may influence physical activity Res Q Exercise Sport 1997 68 345 351 10.1080/02701367.1997.10608015 9421846

[B29] Miller WR Johnson WR A natural language screening measure for motivation to change Addict Behav 2008 33 1177 1182 10.1016/j.addbeh.2008.04.018 18558466

[B30] Hussey MA Hughes JP Design and analysis of stepped wedge cluster randomized trials Contemp Clin Trials 2007 28 182 191 10.1016/j.cct.2006.05.007 16829207

[B31] Sahlqvist S Song Y Bull F Adams E Preston J Ogilvie D Effect of questionnaire length, personalisation and reminder type on response rate to a complex postal survey: randomised controlled trial BMC Med Res Methodol 2011 11 10.1186/1471-2288-11-62 PMC3110121 21548947

[B32] Schall R Estimation in Generalized Linear Models with Random Effects Biometrika 1991 78 719 727 10.1093/biomet/78.4.719

[B33] Hanley JA Negassa A Edwardes MD Forrester JE Statistical Analysis of Correlated Data Using Generalized Estimating Equations: An Orientation Am Journal Epidemiol 2003 157 364 375 10.1093/aje/kwf215 12578807

